# Dienogest in conjunction with GnRH-a for postoperative management of endometriosis

**DOI:** 10.3389/fphar.2024.1373582

**Published:** 2024-03-07

**Authors:** Ying Ma, Wen-Xin Wang, Ye Zhao

**Affiliations:** ^1^ Department of Gynecology, The First Hospital of Shanxi Medical University, Taiyuan, China; ^2^ First Clinical Medical College, Shanxi Medical University, Taiyuan, China

**Keywords:** adverse bleeding pattern, adverse reactions, DIE, dienogest (DNG), gynecology

## Abstract

**Objective:** The aim of this study is to assess the postoperative efficacy of the combined administration of dienogest (DNG) and gonadotropin-releasing hormone agonists (GnRH-a) in patients diagnosed with endometriosis (EMS), while acknowledging the extensive use of DNG in the extended therapeutic management of EMS.

**Methods:** In this retrospective study, a cohort of 154 patients who underwent conservative surgical intervention for EMS were scrutinized. The cohort was stratified into two distinct groups based on their prescribed pharmacological regimens. Group A, 70 patients received postoperative oral administration of DNG at a dosage of 2 mg/day, whereas Group B, 84 patients underwent treatment involving 3 to 4 injections of GnRH-a post-surgery, followed by DNG therapy. Parameters assessed included pelvic pain visual analog scale (VAS) scores, quality of life metrics (EHP-5), and the incidence of adverse reactions within both groups.

**Results:** Both groups exhibited sustained low VAS scores following the prescribed treatments. The predominant occurrence of adverse bleeding patterns manifested predominantly within the initial 6 months of the treatment. Notably, Group B demonstrated a significantly diminished of experiencing frequent and irregular bleeding in comparison to the DNG group (20.0% vs. 8.3%, 12.9% vs. 3.6%, *p* < 0.05). The administration of GnRH-a did not exacerbate the impact on bone health. Subsequent to health promotion interventions, the incidence of weight gain in both groups declined to 7.1% during the 6-month follow-up (*p* < 0.05). Group B exhibited a 100% satisfaction rate with the treatment, concomitant with a noteworthy reduction in EHP-5 scores (*p* < 0.05). Patients with deep infiltrating endometriosis (DIE) nodules displayed marginally higher postoperative VAS scores than their non-DIE counterparts (0.89 ± 0.96 vs. 0.49 ± 0.78). However, with sustained medication use, pain scores within the DIE group exhibited a continual decrease, maintaining a low level of 0.29 ± 0.67 at 12 months and beyond.

**Conclusion:** The short-term adjunctive use of GnRH-a prior to DNG treatment postoperatively in patients with EMS proves efficacious in mitigating early adverse bleeding, enhancing patient adherence, and improving overall quality of life. Notably, this therapeutic approach demonstrates favorable safety profiles and is equally effective in patients with DIE.

## 1 Introduction

Endometriosis (EMS) constitutes a prevalent gynecological condition, afflicting approximately 6%–10% of women within the reproductive age group, often causing both pain and infertility (Zondervan et al., 2018; Calagna et al., 2020). This condition significantly exerts adverse effects on both the physical and mental wellbeing of affected patients, thereby detrimentally influencing their overall quality of life (Kim et al., 2022; Techatraisak et al., 2022).

Among the various pathological presentations of endometriosis, ovarian endometriosis is the most prevalent type, with an average recurrence rate of 20% (ranging from 0% to 89%) within the initial 2 years post-surgery and up to 50% (15%–56%) within 5 years (Ceccaroni et al., 2019). Consequently, addressing the objectives of preventing and minimizing recurrence, while concurrently prolonging periods of freedom from pain, constitute a pivotal challenge in the comprehensive management of EMS. Postoperative implementation of extended, long-term medication regimens has emerged as a necessary approach (Endometriosis Committee et al., 2018). The spectrum of available pharmaceutical interventions encompasses short-acting oral contraceptives (OCs), gonadotropin-releasing hormone analogues (GnRH-a), and progestogens (Capezzuoli et al., 2022). Although OCs are commonly recommended in various clinical guidelines for endometriosis treatment, they are not officially approved for this indication (Casper, 2017). Extending the duration of GnRH-a treatment to 6 months subsequent to conservative surgery for endometriosis has demonstrated a reduced risk of recurrence (Zheng et al., 2016). However, the concomitant induction of a low estrogen state during such treatment periods may engender a bone loss ranging from 4% to 6% (Johnson et al., 2017). Consequently, a short-term application (less than 6 months) is advised, particularly for young women and adolescents who have yet to attain their peak bone density (Collinet et al., 2018). The different options of steroids add-back therapies (progestins and/or estroprogestins) have been proposed in order to reduce the hypoestrogenic side effects. However, the ideal treatment schedule of GnRH-a is still a matter of debate, particularly regarding the most effective add-back combination (Corte et al., 2020).

Dienogest (DNG), characterized as a fourth-generation selective progestogen, manifests anti-proliferative and anti-inflammatory effects, while concurrently moderating reductions in estrogen levels (Sasagawa et al., 2008; Fischer et al., 2011). Studies conducted by Strowitzki et al. have substantiated the efficacy of DNG (at a dosage of 2 mg/day) in alleviating postoperative endometriosis-related pain, comparable to the outcomes observed with GnRH-a (Strowitzki et al., 2010; Strowitzki et al., 2012). A study conducted in China in 2021 reported that a 52-week course of DNG did not significantly impact lumbar spine bone density (Yu et al., 2019). However, the occurrence of abnormal vaginal bleeding in clinical contexts poses challenges to patient compliance during DNG treatment. Consequently, the exploration of strategies to ameliorate adverse reactions during DNG therapy assumes importance, representing an area that warrants further research attention.

In this cohort study we investigated cases of conservative surgery for ovarian endometriosis, employing DNG as a therapeutic modality and incorporating a preliminary phase of short-term GnRH-a therapy prior to initiating DNG. The primary aim is to scrutinize and document the adverse reactions and overall safety of patients during the course of treatment. Given the persistent debate surrounding the efficacy of progestogen therapy in addressing deep infiltrating endometriosis (DIE) nodules, we also systematically assessed the effectiveness of DNG in the treatment of such lesions (Reis et al., 2020).

## 2 Research methods

### 2.1 Patients

Approval for this study was granted by the Ethics Review Committee of the First Hospital of Shanxi Medical University (No. KYLL-2023-124). A retrospective examination was conducted on the medical records of patients who had undergone laparoscopic conservative surgery for ovarian endometriosis at the First Hospital of Shanxi Medical University from January 2019 to December 2022, with follow-up extending until October 2023.

#### 2.1.1 Inclusion criteria

(1) Female patients aged 18–45 years.
(2) Complete case records.
(3) Diagnosis of EMS through laparoscopic exploration and histopathological examination, with subsequent laparoscopic conservative surgery involving lesion excision.
(4) Patients subjected to extended treatment with DNG and GnRH-a post-surgery.

#### 2.1.2 Exclusion criteria

(1) Individuals expressing a wish for conception within the first year following surgical intervention or those currently in the lactation period.
(2) Individuals with a history of vascular thrombosis, liver disease, kidney disease, coagulation disorders, or autoimmune diseases.
(3) Cases presenting with other conditions leading to irregular vaginal bleeding, such as: endometrial polyps, submucosal uterine fibroids, atypical hyperplasia of endometrium, adenomyosis, endometrial cancer, and cervical lesions.
(4) Women in the perimenopausal period: estrogen levels close to menopausal levels, anti-Mullerian hormone (AMH) ≤1.1 ng/mL, and follicle stimulating hormone (FSH) > 25 U/L.
(5) Individuals exhibiting allergies to the medications used in this study.
(6) Individuals with abnormal blood coagulation function and mental, conscious and language disorders.
(7) Individuals with a previous history of depression or mental illness.

### 2.2 Postoperative medication treatment

**FIGURE 1:**
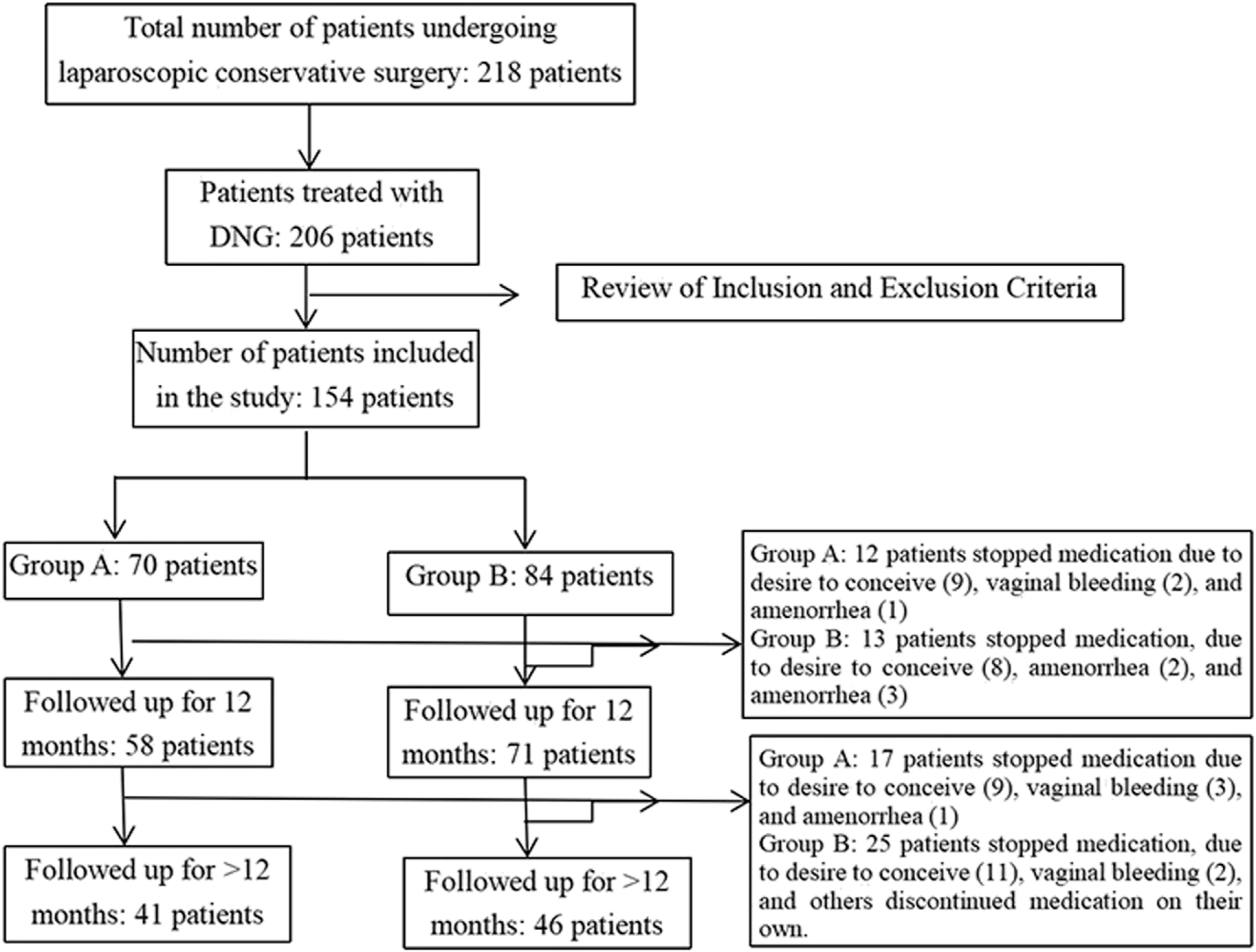
Flowchart of participant follow-up.

**FIGURE 2:**
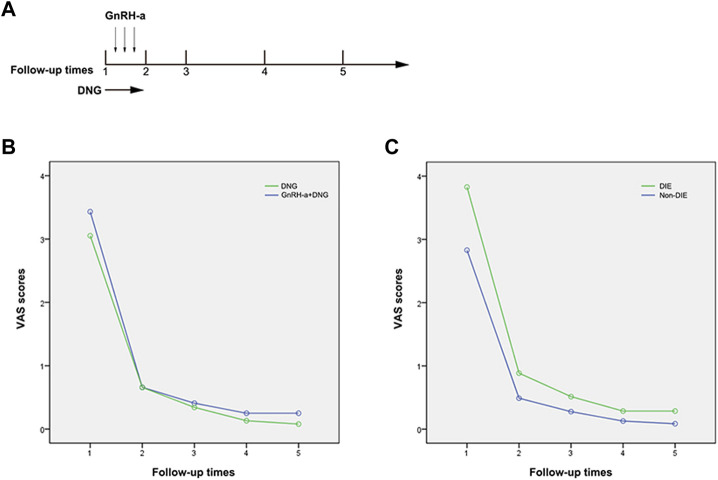
Changes in Pain Scores Preceding and Post Treatment. Note: **(A)** Times of follow-up: 1) pre-surgery first follow-up; 2) second follow-up at 3 months post-surgery; 3) third follow-up at 6 months post-surgery; 4) fourth follow-up at 12 months post-surgery; 5. Fifth follow-up over 12 months post-surgery. **(B)** Changes in pain scores before and after treatment in the DNG group and GnRH-a + DNG group; **(C)** Changes in pain scores before and after treatment in the DIE and non-DIE groups.

Based on the retrospective analysis, a total of 281 patients underwent laparoscopic conservative surgery. Among these, 206 patients received postoperative treatment with DNG, and 154 satisfied the specified inclusion and exclusion criteria. The patients were subsequently stratified into two groups based on their respective medication regimens. Group A received immediate postoperative treatment with DNG (dienogest, Bayer Weimar GmbH und Co. KG, Germany, 2 mg/day), In contrast, Group B 84initiated gonadotropin-releasing hormone agonist (GnRH-a) therapy (subcutaneous injection of leuprorelin acetate microspheres, 3.75 mg, Beijing Bontech) after the first menstrual period, followed by injections every 28 days for 3 to 4 cycles, and subsequently maintained long-term treatment with DNG (2 mg/day). The follow-up status of the included patients is depicted in Figure 1 and postoperative medication treatment is shown in Figure 2A.

### 2.3 Follow-up assessment

Patient data, encompassing obstetric history, past medical history, body mass index (BMI), comorbidities, medication and surgical history, along with the surgical diagnosis of the endometriosis subtype, were systematically collected through the retrospective review of historical medical records. Additionally, the American Society for Reproductive Medicine (ASRM) staging was documented. After initiation of DNG treatment, follow-ups were conducted at three-month intervals, facilitated through either outpatient visits or telephone calls. The medication records of the patients were diligently collected, and instances leading to the discontinuation of medication were comprehensively documented. The process of data collection concluded upon the discontinuation of medication.

#### 2.3.1 Efficacy evaluation

The Endometriosis Quality of Life (EHP-5) score was determined using the EHP-5 questionnaire, which comprises five categories, resulting in a total score range of 0–10 (Harada and Taniguchi, 2010). Elevated scores on this scale signify a diminished quality of life.

The Pelvic Pain Visual Analog Scale (VAS) was used for the evaluation of pelvic pain in all patients, with scores ranging from 0 to 10.

#### 2.3.2 Adverse reactions evaluation

(1) Assessment of Vaginal Bleeding Patterns: The evaluation adhered to the definitions of uterine bleeding as stipulated by the Federation International of Gynecology and Obstetrics (FIGO), with specific details outlined in Table 1 (Fraser et al., 2011).
(2) Evaluation of Additional Adverse Reactions: This encompassed an assessment of various adverse reactions such as breast discomfort, weight gain, depressive moods, insomnia, night sweats, leg pain, hair loss, acne, among others.

**TABLE 1:** Classification of bleeding patterns.

Based on WHO definition	
Favorable bleeding patterns	
Amenorrhea	No bleeding during the reference phase
Rare Bleeding	Bleeding/spotting occurs no more than 3 times
Frequent Bleeding	Bleeding/spotting occurs more than 5 times
Adverse Bleeding Patterns	
Irregular Bleeding	Experience three to five episodes of bleeding or spotting, each lasting for a duration of 14 days or more, and characterized by fewer than three intervals of being free from bleeding
Prolonged Bleeding	A minimum of one occurrence of bleeding or spotting persisting for a duration of 14 days or longer
Acceptable Bleeding	None of the above

Note: Based on WHO, recommendations, 90 days (3 months/3 cycles) constitute one reference period, and 1 year comprises four reference periods.

### 2.4 Sample size calculation and statistical methods

Based on the sample size estimation formula for comparing two independent sample rates,

$$n_{1}=n_{2}=\frac{1}{2}{\left[\frac{z_{ɑ/2}+z_{\beta }}{\arcsin\sqrt{p_{1}}-\arcsin\sqrt{p_{2}}}\right]}^{2}$$

In the formula, n1 and n2 represent the sample sizes required for the two samples, while p1 and p2 denote the estimates of the population rates, respectively. $z_{ɑ/2}$ and $z_{\beta }$ are the z-values of the standard normal distribution corresponding to the test level and type II error β, α = 0.05 (two-sided) and β = 0.20. In the pre-experimental, the incidence of adverse reactions in the DNG group was 66%, in the GnRH-a and DNG group was 43%, and the two groups were enrolled as 1:1. In a further retrospective study, the final sample content of the two groups was 70, indicating that at least 70 cases need to be observed in each group in this study.

The collected data was analyzed using SPSS statistical software version 27.0. Continuous variables that follow a normal distribution, such as age and height, are typically described using the mean ± standard deviation. Comparisons of means between groups were performed using the t-test. Conversely, continuity variables that do not meet the normal distribution, such as BMI, weight, onset time, duration, preoperative CA125 levels, and quality of life scores, were described by the median and interquartile spacing (M, P25, P75). Comparisons of means between groups were performed using the Wilcoxon signed-rank test. The incidence of categorical data between groups was assessed using the chi-squared test, with categorical data including symptoms, adverse reactions, and satisfaction. Pelvic pain (VAS) scores were compared before and after treatment using repeated measures ANOVA. A significance level of α = 0.05 was applied for all tests, with *p* < 0.05 considered indicative of statistical significance.

## 3 Results

### 3.1 General information

A total of 154 patients met the inclusion criteria, with 70 patients comprising Group A (DNG group) and 84 patients constituting Group B (GnRH-a + DNG group). No statistically significant differences in general data between the two groups were observed (*p* > 0.05), ensuring comparability (refer to Table 2 for details).

**TABLE 2:** Comparison of baseline characteristics between Group A and Group B.

General characteristics	Group A (*n* = 70)	Group B (*n* = 84)	Z/t/χ^2^ value	*p*-value
Age (years)	35.21 ± 6.72	33.80 ± 7.02	−1.271	0.206
BMI(kg/m^2^)	21.33 (19.73, 23.88)	20.69 (19.53, 23.55)	−1.236	0.217
Weight (kg)	55.00 (52.00, 62.75)	54.50 (49.25, 6.75)	−1.266	0.206
Height (cm)	161.47 ± 3.19	162.44 ± 3.97	1.647	0.102
Median duration of disease (months)	6.50 (2.75, 12.00)	5.50 (2.0, 12.00)	−0.835	0.404
Median duration of medication use (months)	14.00 (12.00,18.25)	14.50 (12.00, 19.00)	−0.129	0.897
Obstetric history			2.329	0.127
Pregnant	46	45		
Not pregnant	24	39		
The diameter of the cyst (cm)	7.0 (6.0,8.0)	7.0 (6.0,8.0)	−0.972	0.331
Laterality of endometrioma			0.115	0.735
unilateral	56	69		
bilateral	14	15		
algomenorrhea	58	77	2.740	0.098
algopareunia	24	25	0.360	0.548
Median pre-surgery VAS score (points)	3.05 ± 1.99	3.43 ± 1.70	−0.979	0.328
Median pre-surgery EHP-5 score (points)	39.00 (36.00,47.00)	45.5 (36.00,54.00)	−0.923	0.356
Staging (cases)			1.537	0.464
Stage II	20	17		
Stage III	27	38		
Stage IV	23	29		
Pre-surgery CA125	39.00 (20.75, 61.25)	37.00 (24.00, 61.75)	−0.479	0.632
Concurrent DIE (cases)	26	29	0.114	0.736

Note: n, total number. BMI, body mass index.

Specifically, 83 patients received DNG treatment for more than 24 months, and 76 patients continued the treatment for more than 36 months (Figure 1). Notably, during the medication period, only 1 patient in Group B experienced a recurrence of an ovarian cyst at the 12-month follow-up.

### 3.2 Comparison of treatment regimens on pelvic pain management in both groups

Pelvic pain VAS scores were systematically recorded during the follow-up period. In Group A, the recurrence rate was 4.3% (3 out of 70), and in Group B, it was 3.5% (2 out of 84). Notably, the recurrence severity was low, and there was no necessity to escalate analgesic medication. There was no statistically significant difference in the median pelvic pain scores pre-surgery between Groups A and B. At the three-month medical treatment both groups displayed a noteworthy reduction in scores, averaging 0.66 (*p* < 0.001), indicative of comparable surgical efficacy in lesion removal for both groups. At 6 months follow-up, the VAS scores in both groups decreased to 0.34 ± 0.67 in group A and 0.41 ± 0.69 in Group B, with no significant difference between the two groups. This suggests that pain control with GnRH-a before DNG remained stable in the early stage (refer to Table 3). Through continuous monitoring during drug treatment, this effect remained constant throughout the observation period. After more than 12 months of medical treatment, we observed that VAS scores were consistently maintained at a low level for both treatment modalities, with no significant difference between the two groups (0.08 ± 0.27 vs. 0.25 ± 0.62), showing no discernible significant fluctuations (refer to Figure 2B). Therefore, long-term treatment following laparoscopic conservative surgery can effectively improve pelvic pain. The management of pelvic pain with the combination of GnRH-a and DNG demonstrated the efficacy comparable to that observed with DNG alone.

**TABLE 3:** Changes in pain scores preceding and following treatment in both groups.

	Pre-surgery	3 months medication	6 months medication	12 months medication	>1 2 months medication	F	*p*
DNG group	3.05 ± 1.99	0.66 ± 0.97	0.34 ± 0.67	0.13 ± 0.48	0.08 ± 0.27	239.038^a^	<0.001^a^
DNG-GnRH-a group	3.43 ± 1.70	0.66 ± 0.81	0.41 ± 0.69	0.25 ± 0.61	0.25 ± 0.62		
	F = 0.775^b^		*p* = 0.381^b^				

^a^ Note: : a: F and *p*-values pertaining to the time effect and follow-up group, respectively.

^b^ : F and *p*-values associated with the group effect.

### 3.3 Impact of DNG on pain in patients diagnosed with DIE

Among the 154 patients enrolled, 55 were diagnosed with DIE, with 26 belonging to Group A and 29 to Group B. Preoperative pelvic pain exhibited a statistically significant elevation in the DIE group compared to the non-DIE group (*p* = 0.017), indicating a notable difference. Post-surgery, the pelvic pain VAS score in the DIE group demonstrated a significant reduction to 0.89 ± 0.96, in contrast to 0.49 ± 0.78 in the non-DIE group. This suggests slightly less effective postoperative pain relief in patients with DIE. However, with sustained medication usage, the pain scores in the DIE group continued to diminish, maintaining a low level of 0.29 ± 0.67 at 12 months and beyond (refer to Table 4; Figure 2C). This observation underscores the effectiveness of DNG in managing postoperative pain in patients with DIE.

**TABLE 4:** Alterations in pain scores in the DIE and Non-DIE groups preceding and subsequent to treatment.

Group	Pre-surgery	3 months medication	6 months medication	12 months medication	>1 2 months medication	F	*p*
DIE Group	3.83 ± 1.95	0.89 ± 0.96	0.51 ± 0.78	0.29 ± 0.67	0.29 ± 0.67	256.906^a^	<0.001^a^
Non-DIE Group	2.83 ± 1.66	0.49 ± 0.78	0.28 ± 0.58	0.13 ± 0.45	0.09 ± 0.28		
	F 5.92^b^		*p* 0.017^b^				

^a^ Note: : F and *p*-values for the time effect and follow-up group, respectively.

^b^ : F and *p*-values for the group effect.

### 3.4 Comparison of post-treatment bleeding patterns between the two groups

**FIGURE 3:**
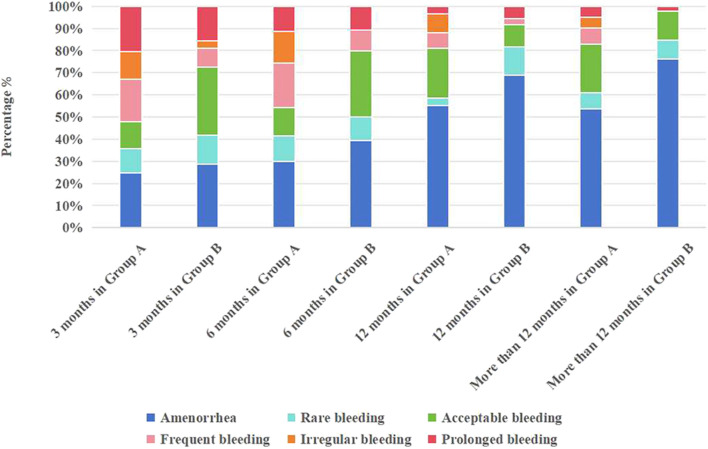
Statistics of bleeding patterns in Group A and Group B.

Most of the patients observed in the study underwent changes in their bleeding patterns. In the initial 3 months of DNG use, the rate of normal/acceptable bleeding in Group B was significantly higher at 31.0% (26 out of 84) compared to Group A, which registered 12.9% (9 out of 70), with *p* < 0.05. No difference in the rate of amenorrhea was observed between the two groups within the initial 6 months of DNG use. However, as the therapy continued, the incidence of amenorrhea gradually increased. After more than a year of therapy, the amenorrhea rate in Group A reached 53.7%, and was even higher in Group B at 73.9% (refer to Table 5; Figure 3).

**TABLE 5:** Statistical analysis of favorable bleeding patterns during treatment in both groups [n (%)].

		Group A	Group B	Value	*p*
3 Months	Amenorrhea	14 (25.7)	24 (28.6)	1.030	0.310
	Rare Bleeding	8 (11.4)	11 (13.1)	0.098	0.754
	Acceptable Bleeding	9 (12.9)	26 (31.0)	7.119	0.008
6 Months	Amenorrhea	21 (30.0)	33 (39.3)	1.446	0.229
	Rare Bleeding	8 (11.4)	9 (10.7)	0.020	0.888
	Acceptable Bleeding	9 (12.9)	25 (29.8)	6.342	0.012
12 Months	Amenorrhea	32 (55.2)	49 (69.0)	2.618	0.106
	Rare Bleeding	2 (3.4)	9 (12.7)	3.485	0.062
	Acceptable Bleeding	13 (22.4)	7 (9.9)	3.841	0.050
>12 Months	Amenorrhea	22 (53.7)	35 (76.1)	4.827	0.028
	Rare Bleeding	3 (7.3)	4 (8.7)	0.056	0.813
	Acceptable Bleeding	8 (22.0)	6 (13.0)	1.206	0.272

At the 3, 6, 12, and over 12 months of follow-up, Group A exhibited adverse bleeding patterns with probabilities of 54.3%, 45.7%, 19.0%, and 17.1%, respectively. In contrast, Group B revealed probabilities of 27.3%, 20.2%, 8.5%, and 4.3%. As depicted in Figure 3, both groups experienced elevated rates of adverse bleeding patterns during the initial 6 months of medication use. Remarkably, at the 3-month follow-up, Group B exhibited significantly lower rates of frequent and irregular bleeding compared to Group A (20.0% vs. 8.3%, 12.9% vs. 3.6%, respectively, *p* < 0.05). By the 6-month follow-up, the rate of prolonged bleeding decreased in both groups, with no irregular bleeding observed in Group B (refer to Table 6). As the use of DNG extended, the bleeding patterns improved, stabilizing after 12 months of medication use (*p* < 0.05).

**TABLE 6:** Statistics of adverse bleeding patterns during treatment in both groups [n (%)].

		Group A	Group B	Value	*p*
3 Months	Frequent Bleeding	14 (20.0)	7 (8.3)	4.413	0.036
	Irregular Bleeding	9 (12.9)	3 (3.6)	4.582	0.032
	Prolonged Bleeding	15 (21.4)	13 (15.5)	0.909	0.340
6 Months	Frequent Bleeding	14 (20.0)	8 (9.5)	3.422	0.064
	Irregular Bleeding	10 (14.3)	0 (0.0)	12.833	<0.001
	Prolonged Bleeding	8 (11.4)	9 (10.7)	0.020	0.888
12 Months	Frequent Bleeding	4 (6.9)	2 (2.8)	1.198	0.274
	Irregular Bleeding	5 (8.6)	0 (0.0)	6.367	0.012
	Prolonged Bleeding	2 (3.4)	4 (5.6)	0.344	0.558
>12 Months	Frequent Bleeding	3 (7.3)	0 (0.0)	3.486	0.062
	Irregular Bleeding	2 (4.9)	0 (0.0)	2.297	0.130
	Prolonged Bleeding	2 (4.9)	1 (2.2)	0.476	0.490

Note: Group A: direct DNG 2 mg/day; Group B: GnRH-a 3.75 mg/course 3-4 times + DNG 2 mg/day. χ^2^ test analysis (*p* < 0.05 indicates statistical significance). n: total number.

### 3.5 Occurrence of other adverse reactions

**FIGURE 4:**
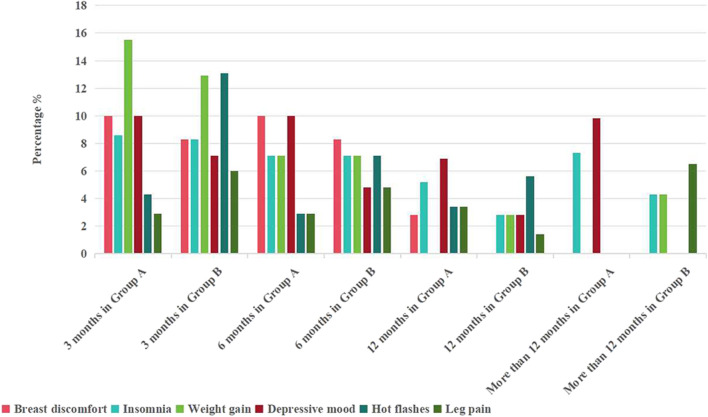
Statistics of adverse reactions.

Some patients encountered one or more adverse reactions early in the treatment process. The data reveals that both groups exhibited a heightened incidence of adverse reactions during the initial 3 months of treatment. These reactions predominantly included weight gain, breast discomfort, and depressive mood. In Group A, 13 patients (15.5%) experienced notable weight gain, compared to 9 patients (12.9%) in Group B, with no statistically significant difference between the groups. Following health education and dietary improvements, a noteworthy reduction in the rate of weight gain was observed at the 6-month follow-up, with 5 cases (7.1%) in Group A and 6 cases (7.1%) in Group B (refer to Figure 4). With ongoing health education and sustained medication use, issues related to insomnia and depressive mood gradually improved in patients. The occurrence of leg pain exhibited no significant variation throughout the follow-up period in either group, and there was no discernible increase in leg pain attributable to the use of GnRH-a, potentially causing calcium loss.

### 3.6 Long-term medication treatment and quality of life improvement

There was no disparity in the quality of life scores between the two groups pre-surgery. Following 6 months of DNG treatment, Group A exhibited a decrease in the quality of life score from 39 to 25, reflecting a reduction of 15 points. In contrast, Group B experienced a more substantial improvement, with a 19-point reduction (*p* < 0.001) (refer to Table 7). Notably, the treatment satisfaction level attained 100% in both groups.

**TABLE 7:** Quality of Life Score M (P25, P75) in Both Groups.

	N	Pre-surgery	6 months medication	Quality of Life Score Changes
Group A	70	39.00 (36.00, 47.00)	25.00 (22.00, 28.00)	15.00 (8.00, 18.25)
Group B	80	41.5 (32.00, 50.00)	19.00 (16.00, 28.00)	19.00 (15.00, 26.00)
Z		−0.846	−3.441	−4.302
P		0.967	0.001	<0.001

## 4 Discussion

EMS is acknowledged as a chronic disease. However, despite notable advancements in related drug treatments in recent years, pain continues to pose a substantial challenge for patients diagnosed with EMS.

### 4.1 DNG for pain control

Several studies have highlighted the considerable impact of DNG in mitigating pelvic pain among patients with EMS. In 2019, following at least 6 months of DNG treatment for 3,356 patients with endometriosis in South Korea, the VAS score significantly decreased by 28.19 ± 28.39 mm (Cho et al., 2020). A Meta-analysis of DNG as maintenance therapy after conservative surgery for endometriosis indicated that DNG maintenance therapy resulted in a significant decrease in VAS scores and a low recurrence rate at 12 months after surgery, compared with LNG-IUS, GnRH-a treatment (Liu et al., 2021), highlighting its efficacy as a postoperative maintenance treatment strategy. Our findings align with existing research, affirming that DNG effectively alleviates postoperative pelvic pain. Furthermore, the combined administration of GnRH-a and DNG was observed to sustain effective pain control following endometriosis surgery, demonstrating non-inferiority compared to the use of DNG alone.

A previous study reported that after 24 months of DNG treatment, the pain recurrence rate in patients was 2.7% (*n* = 4/146), which was three times lower than in the non-intervention group, with a median recurrence time of 22.2 months (Techatraisak et al., 2022). In our study, 76 patients consistently adhered to the administration of DNG for more than 3 years, and only 5 cases experienced dysmenorrhea recurrence during the follow-up, resulting in a recurrence rate of 3.2%. Importantly, the severity of pain was significantly lower than pre-surgery, indicating that DNG is effective in managing EMS-related pain over the long term and can extend the time to recurrence.

### 4.2 Pain analysis in DIE

DIE often involves lesions infiltrating various anatomical structures, including the uterosacral ligament, rectouterine pouch, rectovaginal septum, and even penetrating the vagina. Surgical excision of lesions and adhesion release constitute the primary modalities for addressing pain associated with DIE. However, complete surgical removal of all lesions may not always be feasible. Dai et al. observed a complete excision rate of only 28.6% for rectal DIE, followed by 83.3% for dome-type lesions (Dai et al., 2010). The pain recurrence rate for patients experiencing moderate to severe dysmenorrhea is 7.8% 2 years post-surgery, and for rectal DIE, it is 14.2% (Dai et al., 2012). To date, comprehensive large-scale clinical trials and systematic research on high-risk factors and preventive measures for DIE recurrence are still lacking. Nevertheless, the postoperative use of medication and the long-term management of individuals with DIE are deemed crucial, particularly in primary care settings, where prolonged medication use compensates for the limitations of achieving surgical thoroughness.

Marcello et al. determined that DNG is as effective as GnRH-a in preventing the recurrence of DIE and associated pelvic pain, with better tolerability (Ceccaroni et al., 2021). Continuous oral intake of DNG can effectively improve VAS scores, making DNG a preferred pharmacological option for the long-term management of DIE. In terms of surgical approaches for DIE, more conservative methods, such as bowel lesion excision instead of butterfly excision or partial bowel resection and anastomosis, can be chosen to reduce the occurrence of surgical complications and ensure patient safety.

### 4.3 Changes in vaginal bleeding pattern

Irregular vaginal bleeding during DNG treatment constitutes a significant factor influencing patient compliance, with an incidence rate ranging from 10.4% to 29.4% (Luisi et al., 2015; Uludag et al., 2021; Techatraisak et al., 2022). This phenomenon diminishes treatment satisfaction and prompts some patients to discontinue medication. In the current study, the incidence of adverse bleeding in the first 6 months of DNG treatment alone was 54.3% and 45.7%, with the probability of adverse bleeding pattern period decreasing by 19.0% after 12 months of the treatment. Addressing how to improve the bleeding pattern in EMS patients during the early stage of DNG treatment has been a challenging problem.

We explored the use of GnRH-a prior to long-term oral DNG, capitalizing on the low hormonal levels produced by GnRH-a, which are even lower than those in the direct DNG use group (Schindler et al., 2006). This strategy aims to prevent endometrial proliferation and reduce the risk of adverse bleeding. The combined GnRH-a group exhibited a lower incidence of adverse bleeding in the first two follow-ups post-medication, at 27.3% and 20.2%, which was significantly lower than the DNG-only group. In the combination group, the probability of frequent bleeding and irregular bleeding in the first 3 months and 6 months were 8.3% and 3.6%, respectively, which were significantly lower than those in the single-agent DNG group at 20.0% and 12.9%. This difference was statistically significant. Additionally, the occurrence of amenorrhea was higher in the combination group.

The results indicate that 3-4 injections of GnRH-a before postoperative DNG were effective in reducing adverse bleeding patterns, resulting in enhanced compliance. This approach represents one of the strategies for ensuring long-term drug management. Therefore, in the future clinical practice, promoting the combination of GnRH-a and DNG may improve the quality of life for patients and increase their satisfaction. However, since amenorrhea can pose a significant psychological burden, it is imperative to comprehensively explain the anticipated changes in bleeding patterns to patients, to alleviate their anxiety.

### 4.4 Other adverse reactions

The reduction in peripheral blood estrogen levels induced by DNG significantly diminishes the risk of bone loss. Ota et al. (Ota et al., 2021) reported, there was no significant difference in bone turnover after 3 months of DNG treatment in young women. Additionally, according to Ebert et al. (Ebert et al., 2017), there was a partial recovery in lumbar spine BMD after the 52-week therapy for teenage endometriosis with DNG 2 mg. Observations made over a period of up to 3 years after initiating DNG treatment did not reveal an increase in symptoms such as leg discomfort. Furthermore, short-term use of GnRH-a for 3 months did not elevate the risk of bone loss, affirming the safety of this regimen in terms of its impact on bone health.

The impact of DNG on weight tends to stabilize after 24 weeks of treatment (Lang et al., 2018). In our study, following health education for patients, the change in weight was notably reduced 6 months after commencing medication. 6.9%–10.0% of patients experienced depressed mood, and no safety signal regarding serious adverse events were observed. Only one patient discontinued medication due to breast pain, and one due to acne. Patient satisfaction with quality reached 100%. Therefore, DNG demonstrates a favorable safety profile and tolerability in the long-term postoperative drug management process.

### 4.5 Limitations of this study

In the context of DNG treatment, using leg pain as the sole indicator to monitor bone loss may underestimate the true prevalence of adverse reactions. Furthermore, this retrospective clinical study is subject to variations in prescribing decisions and patient compliance by clinicians, potentially introducing biases into the results. Future research efforts should include prospective controlled studies with larger sample sizes to validate treatment effectiveness in real-world settings using enhanced research methodologies.

## 5 Conclusion

Conservative surgery for endometriosis, coupled with DNG treatment, proves highly effective in substantially alleviating pain for patients, demonstrating clear efficacy, especially in those with DIE. The short-term adjunctive therapy with GnRH-a serves to alleviate early adverse bleeding related to treatment, enhance the quality of life of patients and enhance overall compliance. Consequently, it is strongly recommended that patients adhere to long-term medication, with a focus on health education throughout the treatment period.

## Data Availability

The original contributions presented in the study are included in the article/Supplementary material, further inquiries can be directed to the corresponding author.
